# A new role for *Hif-1α*

**DOI:** 10.7554/eLife.82028

**Published:** 2022-08-30

**Authors:** Mingyu Shin, Jiwon Shim

**Affiliations:** 1 Department of Life Sciences, Hanyang University Seoul Republic of Korea; 2 Research Institute for Natural Science, Hanyang University Seoul Republic of Korea; 3 Hanyang Institute of Bioscience and Biotechnology, Hanyang University Seoul Republic of Korea

**Keywords:** animal flight, hypoxia inducible factor, energy metabolism, dj-1, alternative splicing, redox homeostasis, Other

## Abstract

A gene normally involved in responding to hypoxia helps to protect insect muscles during migratory flight in a non-oxygen dependent manner.

**Related research article** Ding D, Zhang J, Du B, Wang X, Hou L, Guo S, Chen B, Kang L. 2022. Non-canonical function of an Hif-1α splice variant contributes to the sustained flight of locusts. *eLife* **11**:e74554. doi: 10.7554/eLife.74554.

Animals have evolved complex respiratory systems which efficiently deliver oxygen to every part of their body. Still, bursts of oxygen shortages can occasionally take place when metabolic needs surpass supply. Long-distance flying, for example, requires desert locusts to consume 30–150 times more oxygen than they do at rest (Armstrong and Mordue, 1985).

In response, cells can recruit hypoxia-inducible factors – or Hif, for short – which are formed of combinations of Hif-α and Hif-ß modules. These highly conserved proteins can bind to genetic sequences (known as hypoxia-response elements) to regulate genes that control how cells and organisms adjust to a lack of oxygen (Schofield and Ratcliffe, 2004). Under normal conditions, Hif proteins undergo oxygen-dependent chemical modifications that lead to their degradation (Ivan et al., 2001; Min et al., 2002). Low levels of oxygen inhibit this process, stabilising the proteins and activating various hypoxia-related genes (Wenger et al., 2005).

Overall, regulating the activity of Hif proteins – and especially which genes they target – involves a wide range of pathways, molecules, and binding partners (Chandel et al., 2000; Guzy et al., 2005). These processes can even, on occasion, be independent of oxygen levels, allowing Hif proteins to participate in other types of life processes, such as the development of insect blood cells (Mukherjee et al., 2011).

In addition, several versions of the protein can exist within an organism. Some emerge from closely related Hif genes, but others are isoforms, being created from the same gene through various mechanisms. Now, in eLife, Le Kang, Bing Chen and colleagues at the Chinese Academy of Science and Hebei University – including Ding Ding as first author – report how an isoform of the *Hif-1α* gene contributes to the integrity and performance of insect muscles during flight (Ding et al., 2022).

The team focused on migratory locusts (*Locusta migratoria*), an agricultural pest that can fly hundreds of kilometers per day. Like all invertebrates, these insects express only one *Hif-α* gene, *Hif-1α*. Examining the entire coding sequence of this gene revealed that it leads to the production of two distinct isoforms (Hif-1α1 and Hif-1α2) via a process known as alternative splicing. This mechanism involves the cells reshuffling the coding elements present in the RNA transcripts of the *Hif-1α* gene, resulting in different proteins.

Ding et al. showed that in contrast to Hif-1α1 (which is only detectable under hypoxic conditions), Hif-1α2 lacks the domain required to respond to oxygen levels (Figure 1A). The isoform is abundantly expressed in flight muscles, regardless of oxygen concentration. Interestingly, the experiments showed that Hif-1α2 is essential for the prolonged flight performance of locusts, whereas Hif-1α1 has no effect on this trait.

**Figure 1.**
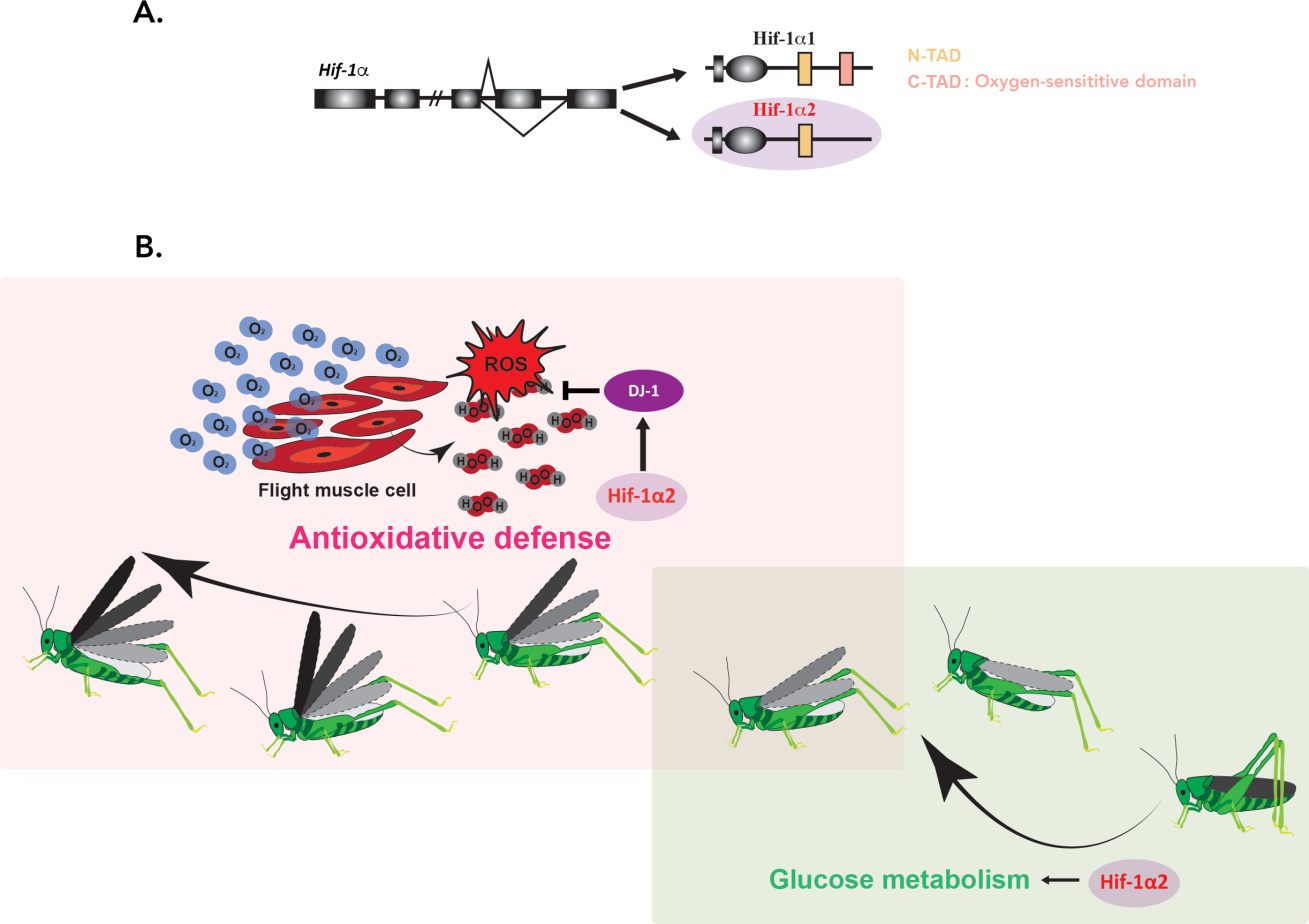
Hif-1*α*2 is required for long-term flight in migratory locusts. (**A**) In migratory locusts, *Hif-1α* produces two distinct isoforms, Hif-1α1 and Hif-1α2 (lilac). Examining the genetic sequence of the two isoforms showed that, unlike Hif-1α1, Hif-1α2 does not carry the oxygen-sensitive C-TAD domain (peach) and it is therefore oxygen-insensitive. Hif-1α2 is expressed specifically in flight muscles. (**B**) During the initial stage of a flight (right; soft green), glucose metabolism is required to meet the need for high energy demands. Hif-1α2 (lilac) upregulates genes involved in this process, but its removal did not impact long-term flight behavior. As the insects continue to fly (left; soft pink), increased oxygen consumption (blue O_2_ molecules) and energy generation lead to an accumulation of harmful reactive oxygen species (ROS) such as hydrogen peroxide (H_2_O_2_; in red and grey). Hif-1α2 helps to deal with these dangerous metabolic byproducts by promoting the expression of the ‘ROS quencher’ DJ-1 (purple), which participates in the antioxidative defense. Animals lacking Hif-1α2 or DJ-1 therefore accumulate ROS in their flight muscles and are unable to fly for an extended period.

The team then genetically manipulated locusts to silence the Hif-1α2 isoform, before examining gene expression profiles in these mutants and in normal insects. This highlighted 12 genes that were significantly downregulated when the isoform was knocked down. Amongst these, 11 are involved in the same energy-creating pathway; however, interfering with this molecular cascade had no discernible effects on the insects’ ability to perform long-term flights. As a result, Ding et al. propose that the pathway plays a role earlier on, as the locust takes off and starts to fly.

The remaining gene, which codes for the DJ-1 protein, helps to protect the organism against reactive oxygen species (or ROS). This class of harmful molecules is released by cellular activity. In flight muscles, their constant presence is correlated with high oxygen consumption, and increases when Hif-1α2 is knocked down. In addition, Ding et al. showed that DJ-1 is enriched in flight muscles; if removed, flight-induced ROS levels soar up and performance becomes impaired.

Finally, the team confirmed a new, non-canonical target for Hif-1α2 by showing that the isoform binds to the hypoxia-response elements present in the promoter of the *DJ-1* gene, even when oxygen levels are normal (Figure 1B). Overall, Ding et al. propose that Hif-1α2 confers a physical advantage in prolonged flight by alleviating the damage linked to ROS while simultaneously maintaining efficient energy production during the initial stage of flight.

Insects were among the first animals to fly, but while some have evolved to be one the most efficient creatures to have taken to the skies, others are far less skilled. The oxygen-sensitive domain of *Hif-1α* has been under high selective pressure throughout evolution; the presence of a range of isoforms for this gene, including oxygen-insensitive variants, is likely to contribute to this divergence in flight performance (Graham and Presnell, 2017). Future studies should investigate exactly how Hif-1α2 is alternatively spliced in flight muscles and how it is controlled independently of oxygen, both in locusts and in other insects. A better grasp of the remarkable versatility of the Hif pathway, including in humans, could help to pinpoint the evolutionary and ecological significance of these genes, and why certain Hif proteins are involved in developmental conditions or cancer.
